# SOCIAL CONNECTIONS AND FALLS AMONG OLDER WOMEN WITH AND WITHOUT HIV IN THE UNITED STATE

**DOI:** 10.1093/geroni/igae098.4078

**Published:** 2024-12-31

**Authors:** Lien Quach, Patricia Lloyd, Kristine M Erlandson, Tracey Wilson, Anna Rubtsova, Anjali Sharma, Jeffrey Burr, Mark Siedner

**Affiliations:** University of Massachusetts Boston, Boston, Massachusetts, United States; University of Massachusetts Boston, Boston, Massachusetts, United States; University of Colorado - Anschutz Medical Center, University of Colorado Anschutz Medical Campus, Colorado, United States; Downstate Health Sciences University, Brooklyn, New York, United States; Rollins School of Public Health, Emory University, Atlanta, Georgia, United States; Albert Einstein College of Medicine, Bronx, New York, United States; University of Massachusetts Boston, Boston, Massachusetts, United States; Massachusetts General Hospital, Boston, Massachusetts, United States

## Abstract

Falls affect one in four older adults annually and are linked to biological and environmental risk factors. Women with HIV (WWH) may be particularly vulnerable due to lower social support and higher loneliness but their relationship with falls is not well understood. We analyzed data from 1,068 women aged 50 and older in the 2017 Women’s Interagency HIV Study (WIHS). Social connections were evaluated using the Multidimensional Social Support Scale, which queries respondents about issues such as support for sharing private worries and help with medical appointments. Loneliness was defined as a score of 6-9 on the UCLA Loneliness Scale (range 0-9). Logistic regression models estimated associations between social connections and loneliness with falls, adjusting for age, education, alcohol use, smoking, and total of comorbidities. Falls were reported by 31.5% of participants, with similar rates between WWH and women without HIV (WWoH) (adjusted odds ratio [aOR]=1.08; 95%CI 0.79,1.49). Loneliness was associated with a higher likelihood of falls (aOR=1.28; 95%CI 1.04,1.82). Some social connection aspects, such as having someone with whom to provide information or share worries (aOR=0.90; 95%CI 0.81, 0.996), or to receive assistance with medical appointments (aOR=0.89; 95%CI 0.80, 0.989), was associated with a lower odd of falls. WWH experience falls at rates similar to WWoH. However, loneliness and lack of social connections are key contributors to falls in older women in the US. Future research should explore whether interventions to mitigate loneliness and enhance social connections impact falls in older women in the US.

